# Biopesticidal Properties of the Probiotic *Brevibacillus laterosporus* Strain B.O.D.

**DOI:** 10.3390/toxins18060251

**Published:** 2026-05-31

**Authors:** M. Florencia Gil, Alessia Vinci, Manuela Casada, Luca Ruiu

**Affiliations:** Department of Agricultural Sciences, University of Sassari, 07100 Sassari, Italy; mfgil@uniss.it (M.F.G.); avinci1@uniss.it (A.V.); m.casada@phd.uniss.it (M.C.)

**Keywords:** insecticidal proteins, entomopathogen, biological control, sequencing, comparative genomics, phylogenesis

## Abstract

*Brevibacillus laterosporus* strain B.O.D. is a well-established commercial probiotic and antimicrobial microorganism that finds use in human health and in agriculture as a biofertilizer. On the other hand, while *B. laterosporus* is a well-known entomopathogenic species, the possible insecticidal potential of strain B.O.D. remains unexplored. To address this knowledge gap, this study combined genome sequencing and comparative analysis with other *B. laterospours* strains and insect bioassays. The genome of *B. laterosporus* B.O.D. was found to harbor a wide range of genes related to entomopathogenicity encoding putative proteases, chitinases, collagenase-like proteases, mosquitocidal proteins, bacillolysin, and spore-surface proteins. Antimicrobial compounds such as gramicidin and surfactin were also found. Sequence alignment with other well-characterized *B. laterosporus* strains and analysis revealed significant differences, which support the corresponding differences in insecticidal activity observed when comparing strain B.O.D. with others against a variety of lepidopteran and dipteran pest species. This study reports for the first time the genome of strain B.O.D., providing a comparative analysis and highlighting its insecticidal properties, which appear more moderate compared to previously characterized entomopathogenic strains of the same species. Everything considered, *B. laterosporus* strain B.O.D. appears to be remarkably versatile, underscoring wide biotechnological potential.

## 1. Introduction

*Brevibacillus laterosporus* is a spore-forming bacterial species with a distinctive spore shape, having a lateral position in the sporangium due to the presence of a canoe-shaped parasporal body attached to one side of the spore coat [1]. Beyond this unique morphology, this species appears to have evolved multifunctional roles in nature, leveraging its probiotic, antimicrobial, biofertilizer, and biopesticidal potential [2]. Accordingly, for instance, strains B.O.D. [3,4], PBC01 [5], and BL1 [6] show probiotic properties contributing to maintaining human and animal gut flora balance and favoring growth and health, while strains OSY-I [7], TSA31-5 [8], and GI-9 [9] produce antibiotics conferring strong antimicrobial properties against human pathogens, and strains BPM3 and A060 are active against plant pathogens [10]. On the other hand, strains K75 [11] and YS-13 [12] were reported to be plant growth promoters. Furthermore, several strains of *B. laterosporus*, such as strains 615 and 621 [13], strain UNISS 18 [14], and strains LMG 15441 and SAM19 [15], have been studied to highlight their insecticidal properties. Although such functional diversity is often linked to specific strains, several traits are highly conserved across the species [16,17], which may enable the discovery of multifunctional strains or strains initially thought to serve a single purpose but possessing broader capabilities. Many of these properties, particularly the insecticidal potential of entomopathogenic bacteria, are related to the insecticidal proteins they produce, which belong to a wide variety of classes [18].

Although the mechanism of the insecticidal action of *B. laterosporus* is not fully understood, and differences between strains are expected, several studies have demonstrated an insecticidal effect resulting from the ingestion of spores or cells. Spores, in particular, appear to be the most active bacterial component; their ingestion leads to the development of ultrastructural damage to the intestinal epithelium, including alterations in microvilli, the appearance of vesicles in the cytoplasm, and ultimately cell lysis. This is followed by deterioration of the intestinal mucosa, leading to paralysis and death of the host [19]. This activity appears to be mediated by bacterial virulence factors, including enzymes such as chitinases and proteases, Mpp toxins, proteins of the spore coat-canoe-shaped parasporal body complex (SC-CSPB), and S-layer proteins of the vegetative cells [17,20,21].

*Brevibacillus laterosporus* strain B.O.D. is a well-established commercial probiotic known for its effectiveness against human microbial pathogens, including fungal species in the *Candida* genus, according to documented antimicrobial properties [3]. This strain is also known for its probiotic benefits on human gut health through antimicrobial and immunomodulatory activities [22]. Accordingly, the safety of this strain for human use can be inferred from its deployment in the commercial drug Flora Balance^TM^ as a spore-based formulation to be taken orally [4].

Beyond these roles in human health, *B. laterosporus* strain B.O.D. has been evaluated for use in agriculture as a biofertilizer and against plant pathogens [3]. Consistent with findings from other *B. laterosporus* strains known to produce a diverse array of bioactive compounds, such as antibiotics and enzymes, strain B.O.D. may also be remarkably versatile, underscoring a wider biotechnological potential. At the species level, *B. laterosporus* exhibits a rich repertoire of antimicrobial and insecticidal compounds, which raises the possibility that strain B.O.D. may also harbor such biosynthetic potential [16]. Although the use of *B. laterosporus* strain B.O.D. is established in agriculture for its antimicrobial and biofertilizing properties [23], its insecticidal potential remains unexplored. Moreover, according to the available public scientific literature, B.O.D. itself has not yet undergone comprehensive genomic or functional characterization.

Therefore, this study aims to address this knowledge gap by combining genome sequencing and comparative analysis with insect bioassays. Accordingly, we (i) sequenced the whole genome of strain B.O.D. and identified genes related to the biopesticidal potential and (ii) evaluated the insecticidal and antimicrobial properties via laboratory bioassays. This dual approach clarifies that the known probiotic, antimicrobial, and biofertilizer functions of strain B.O.D. conceal an underrecognized biopesticidal potential, thereby enhancing our understanding of the versatility and practical applications of this commercially significant strain while also contributing to a broader understanding of the ecology of this bacterial species.

## 2. Results

### 2.1. Brevibacillus laterosporus Strain B.O.D. Genome

**Figure 1:**
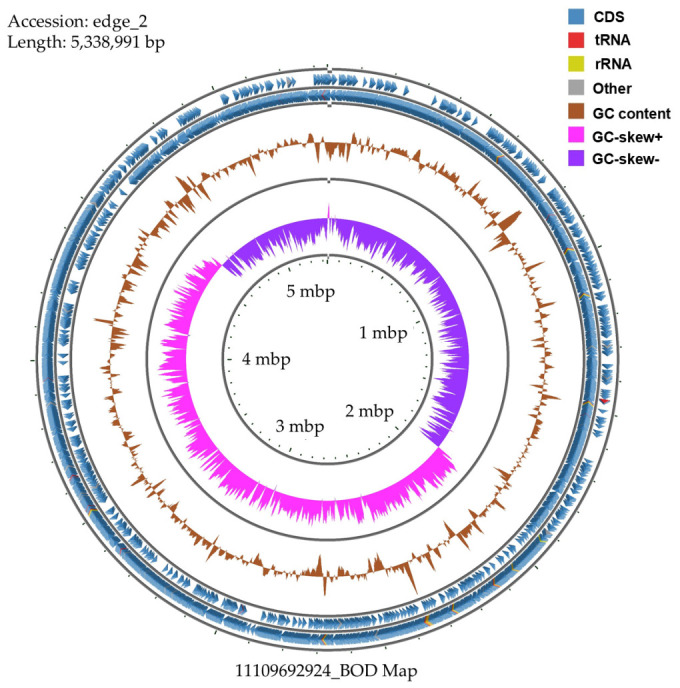
Circular genome map of *B. laterosporus* strain B.O.D. generated with CGView v2.0.3 to illustrate genomic features, GC skew, and sequencing coverage [25]. The two outer rings depict coding regions on the forward and reverse strands, as well as tRNA, rRNA, and other annotated features, as indicated in the legend. The GC content track shows the deviation from the genome-wide average, while the GC skew track provides insights into the replicon’s replication origin and terminus, supporting assessment of assembly completeness.

According to long-read sequencing, *B. laterosporus* B.O.D. whole genome assembly resulted in two contigs, corresponding to the chromosome (5,325,807 bp) and a plasmid (13,184 bp), respectively, for a total assembly length of 5,338,991 bp. The assembly has a GC content of 40.41% and was generated from 57,118 reads totaling 287.26 Mb, with an estimated coverage depth of 54×. Annotation of the genome revealed 112 tRNAs, 36 rRNAs, 20 ncRNAs, 4793 protein-coding sequences (CDSs), and 22 pseudogenes. The circular map of the *B. laterosporus* B.O.D. genome is shown in Figure 1. Several genes putatively involved in the insecticidal action were found in the genome of this *B. laterosporus* strain. These included genes encoding proteases, such as chitinase A (ChiA), chitodextrinase (ChiD), collagenase-like protease (PrtC), and thermophilic serine proteinase (Tsp). Among other factors contributing to virulence against insects, spore surface protein A (CpbA), exosporium protein C (ExsC), and cell wall proteins were identified. The gene encoding the insecticidal protein Mpp75Ab1 was also detected.

Moreover, compounds with antimicrobial action, such as gramicidin and surfactin, were included. Polyketides such as basiliskamide types A and B; non-ribosomal peptide synthetase clusters encoding bogorol A, brevibacillin, surfactin, gramicidin, and ulbactin; and the siderophore petrobactin were identified with AntiSMASH [24].

The genome sequence of *B. laterosporus* strain B.O.D. has been deposited in the NCBI database under accession number JBWXXF000000000.

### 2.2. Genomic Comparisons

**Figure 2:**
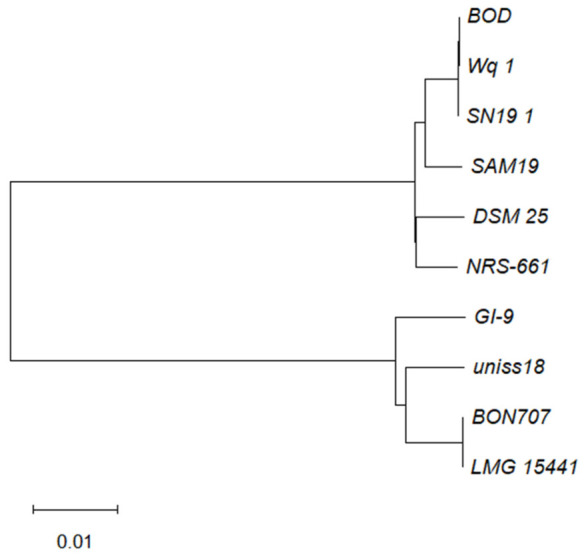
Neighbor-Joining tree of different *B. laterosporus* strains based on whole-genome Average Nucleotide Identity (ANI) values. The tree illustrates the clustering of closely related strains.

According to ANI calculations, *B. laterosporus* strain B.O.D. presents a high similarity with strains Wq_1 and SN19_1 (>99%), suggesting that these isolates actually correspond to the same strain. Strains NRS-661 and DSM_25 were similar to each other and close to strain B.O.D. (>98%). On the other hand, strains LMG_15441, BON707, UNISS18, and GI-9 clustered far from the other strains with less than 90% similarity. Genomic relationships are represented by a Neighbor-Joining tree constructed based on the ANI matrix. Overall, the combined ANI and phylogenetic clustering provide a clear view of genomic similarity and divergence patterns among the various *B. laterosporus* strains (Figure 2 and Table A1).

### 2.3. Multi-Gene Phylogeny

**Figure 3:**
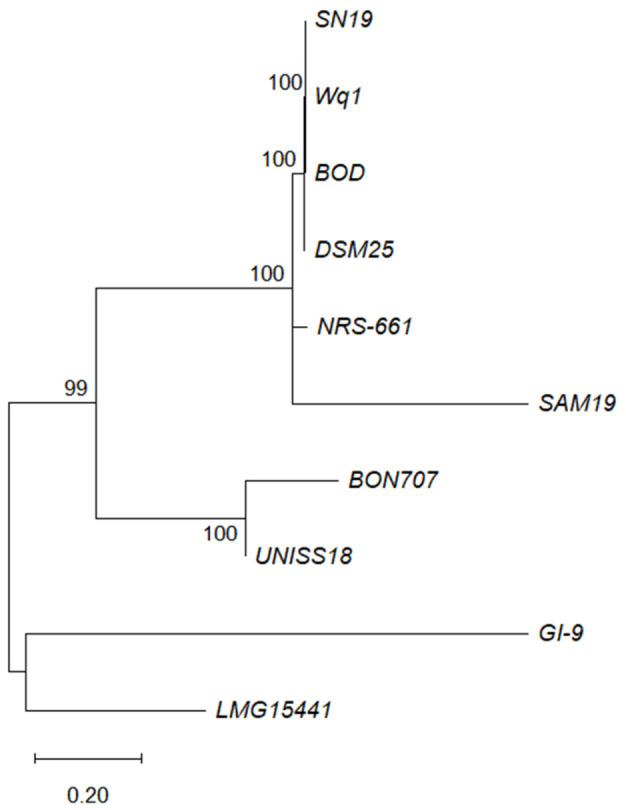
Maximum likelihood phylogenetic tree of different *B. laterosporus* strains based on a multi-gene alignment of 13 concatenated pesticidal gene sequences. The percentage of replicate trees in which the associated taxa clustered together (1.000 replicates) is shown next to the branches. The analysis was performed using MEGA 12 [26,27], and the genes included are listed in Table A2.

A phylogenetic tree was constructed using concatenated sequences of genes associated with biopesticidal activity from different *B. laterosporus* strains (Figure 3). The sequences of these genes in the *B. laterosporus* strain B.O.D. have been deposited in NCBI, and the corresponding accession numbers are provided in Table A2, along with the percentage identity of homologous sequences in other strains relative to B.O.D. Similar to the ANI analysis, strains B.O.D., Wq_1, and SN19_1 consistently group together, while strains SAM19, NRS-661, and DSM-25 are located in sub-branches. Strains LMG_15441 and GI-9 group together with UNISS18 and BON707 in the same cluster.

### 2.4. Insect Bioassays

Bioassays comparing the entomopathogenic activity of *B. laterosporus* strains B.O.D. and UNISS18 against insects of the orders Lepidoptera and Diptera showed that strain B.O.D. exhibits a wide range of insecticidal activity, being slightly less toxic than strain UNISS18 against *L. dispar* (F_2,21_ = 148.8; *p* < 0.001), *H. armigera* (F_2,21_ = 503.4; *p* < 0.001), *M. domestica* (F_2,21_ = 447.5; *p* < 0.001), *L. caesar* (F_2,21_ = 216.9; *p* < 0.001), *A. albopictus* (F_2,21_ = 544.7; *p* < 0.001), and *C. pipiens* (F_2,21_ = 1030.0; *p* < 0.001) (Table 1).

**Figure 4:**
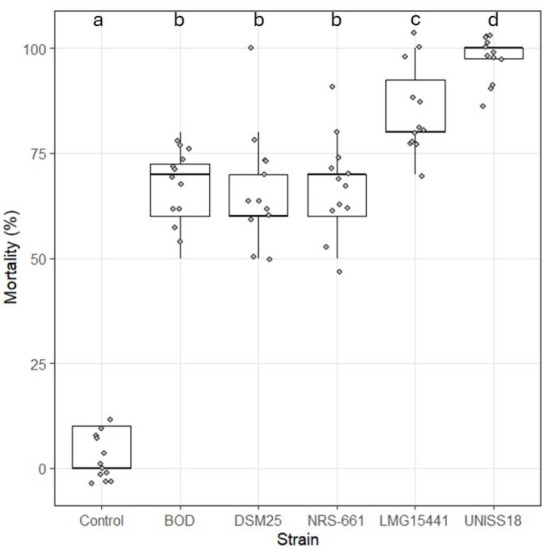
Comparative mortality (mean ± SE) of *M. domestica* adults exposed to a standard concentration (10^8^ spores/mL) of different *B. laterosporus* strains. Different letters above bars indicate significantly different means (1-way ANOVA followed by Tukey’s post hoc test, *p* < 0.05).

To further investigate the effect of strain B.O.D. against *Musca domestica*, in comparison to other *B. laterosporus* strains, bioassays were performed with a standard concentration of 10^8^ spores/mL. Results showed that strain B.O.D. has a moderate toxicity comparable to NRS-661 and DSM25 strains, while strains UNISS18 and LMG15441 are more virulent (F_5,66_ = 137.6; *p* < 0.001) (Figure 4).

**Figure 5:**
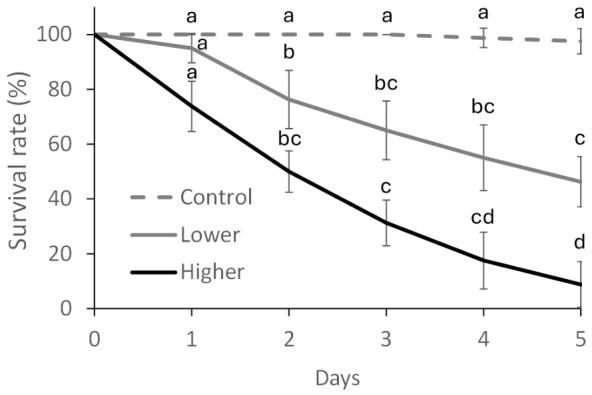
Time-course survival rate (mean ± SE) of *M. domestica* adults exposed to higher (10^9^ spores/mL) and lower (10^7^ spores/mL) concentrations of *B. laterosporus* strain B.O.D. Different letters indicate significant differences (linear mixed-effects model (REML) with Tukey-adjusted pairwise comparisons; *p* < 0.05).

For a more in-depth evaluation of the effects of strain B.O.D. on *M. domestica*, adults were exposed to different concentrations of spore suspension, and mortality over time was registered. Both higher (10^9^ spores/mL) and lower (10^7^ spores/mL) concentrations had negative effects on the survival of *M. domestica* adults after 5 days of treatment (F_2_,_27_ = 24.23, *p* < 0.001). The higher concentration resulted in faster mortality, reaching approximately 50% by day 2 and 90% by day 5, while the lower concentration reached 50% mortality only after 5 days (Figure 5).

**Figure 6:**
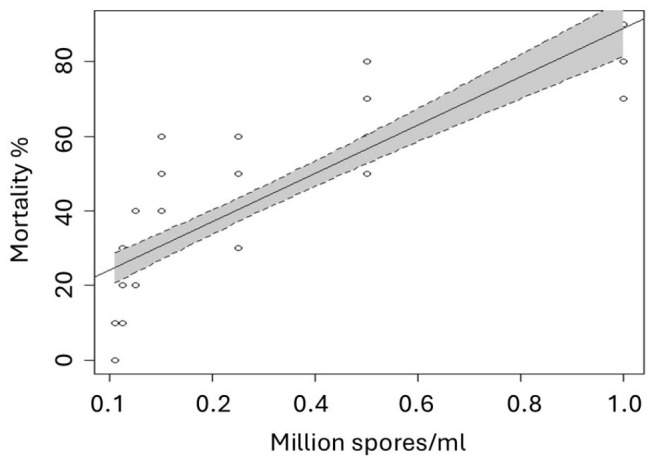
Linear regression plot with 95% confidence intervals (shaded areas) illustrating the predicted relationship between *A. albopictus* larval mortality and *B. laterosporus* B.O.D. spore concentration.

Entomopathogenic activity of strain B.O.D. against Dipterans was further assessed by studying its toxicity against *A. albopictus* larvae exposed to different spore concentrations. Linear regression was significant (F_1,82_ = 184.0, *p* < 0.001, R^2^ = 0.692), with the concentration of *B. laterosporus* strain B.O.D. spores showing a positive effect on *A. albopictus* larval mortality (β = 6.49 × 10^−5^ ± 4.78 × 10^−6^ SE, t = 13.57, *p* < 0.001) (Figure 6). According to probit analysis, the estimated median lethal concentration (LC_50_) was 1.83 × 10^5^ spores/mL (95% CI: 9.86 × 10^4^–3.39 × 10^5^ spores/mL) (β = 1.14 ± 0.25 SE, z = 4.55, *p* < 0.001).

### 2.5. Antifungal Bioassays

**Figure 7:**
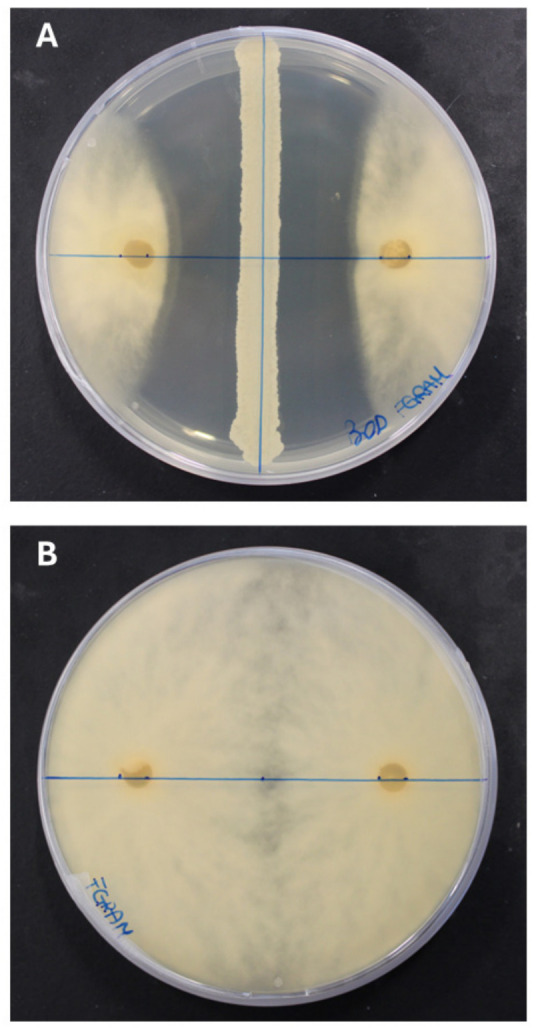
Growth inhibition of *F. graminearum* on PDA plates by *B. laterosporus* strain B.O.D.: (**A**) compared to the untreated control; (**B**) the bacterial streak is positioned in the center of the plate, where two fungal plugs were placed 1.5 cm from the plate’s edge.

**Figure 8:**
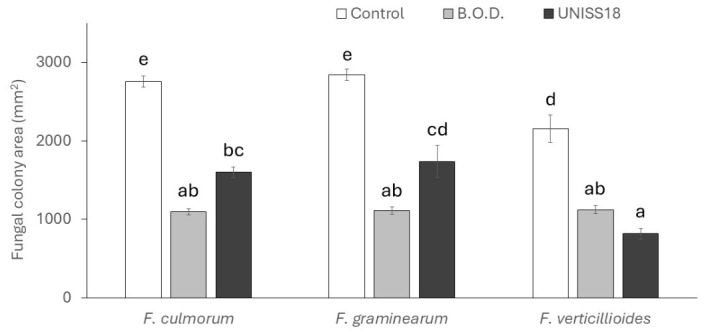
Effect of different *B. laterosporus* strains on the growth of diverse *Fusarium* species on LB agar plates. Bars represent mean ± SE. Different letters indicate significant differences among groups (two-way ANOVA followed by Tukey’s post hoc test, *p* < 0.05).

The antimicrobial properties of *B. laterosporus* strain B.O.D. in comparison with strain UNISS18 were assessed in vitro on fungal phytopathogens of different species (*Fusarium culmorum*, *Fusarium graminearum*, and *Fusarium verticillioides*). For this purpose, the inhibitory effects of bacterial colonies streaked at the center of potato dextrose agar (PDA) plates were estimated by measuring the growth area of each fungus inoculated as a mycelial plug (Figure 7). Both *B. laterosporus* strains showed a significant inhibitory effect compared to the control (F_2,43_ = 160.9, *p* < 0.001), with strain B.O.D. showing a significantly stronger action on *F. graminearum* (F_2,43_ = 16.4, *p* < 0.001) (Figure 8).

## 3. Discussion

*Brevibacillus laterosporus* is a ubiquitous bacterial species known for producing different bioactive compounds across diverse strains. Several studies have highlighted the role of *B. laterosporus* as a probiotic, fertilizer, and biocontrol agent against bacteria, fungi, and invertebrates harmful to animals and plants. Although this species is quite versatile, phylogenetic studies show that specific phenotypic traits are not conserved across strains. Accordingly, while genomes might share some putative toxicity-related genes, many specific genes are found in limited strains [17].

*B. laterosporus* B.O.D. has been commercialized as a probiotic product (i.e., Flora-Balance^™^, Latero-Flora^™^) for more than 25 years [5]. Its antimicrobial and biofertilizer properties are also known [3]. Nevertheless, traits potentially related to the insecticidal activity of this strain have not yet been investigated, thus overlooking its entomopathogenic potential.

In the present study, genome sequencing of *B. laterosporus* B.O.D., followed by whole-genome Average Nucleotide Identity (ANI) comparison with other strains, was performed. Typically, genomes within the same species have an ANI value above 95%, while genomes from different species usually have an ANI value below 90%, although this “between-species” cutoff might change in each genus [28]. ANI calculations showed a close relationship (>99.99%) to strains Wq-1 and SN19-1, clearly indicating that these isolates from China are likely to be the same strain. Both Wq-1 and SN19-1 have been reported to produce antimicrobial compounds, specifically bacteriostatic substances [29,30]. These two strains were isolated from environmental sources, and given their similarity to strain B.O.D., we cannot exclude the possibility that they may have been isolated from soils in which strain B.O.D. was applied as a biofertilizer.

The expected antimicrobial activity of B.O.D. [3] was experimentally confirmed in this study, highlighting its potential against plant pathogenic fungi and aligning with the broad-spectrum antimicrobial properties of the species [2]. Accordingly, genome annotation revealed that it harbors a wide arsenal of putative genes encoding potential antimicrobial compounds. Consistently, non-ribosomal peptide synthetase clusters encoding bogorol A, brevibacillin, surfactin, gramicidin, ulbactin, and petrobactin were detected. Bogorols present a broad-spectrum activity against pathogenic fungi, in addition to their well-known antibacterial properties. Bogorol A is an antibacterial peptide originally isolated and identified from the marine bacterial isolate *B. laterosporus* PNG276 and has been reported to be useful against methicillin-resistant *Staphylococcus aureus* (MRSA) strains and vancomycin-resistant *Enterococcus* (VRE) [30], while Bogorol B, produced by *B. laterosporus* JX-5, is highly active against *F. oxysporum* [31]. Studies reported by Yang [32] and Wu [33] demonstrated that the antimicrobial lipopeptide brevibacillin, produced by different isolates of *B. laterosporus*, showed antimicrobial activity against Gram-positive, Gram-negative bacteria, and fungal species in the genera *Fusarium* and *Aspergillus*. Surfactin, mainly produced by *Bacillus* isolates, has been extensively reported as an efficient antifungal compound with inhibitory effects against phytopathogenic fungi belonging to the genus *Fusarium* [34,35,36,37]. Petrobactin is an iron-scavenging siderophore that has usually been associated with *Bacillus anthracis* str. Sterne virulence. Nevertheless, both pathogenic and non-pathogenic isolates of the *Bacillus cereus* group can produce petrobactin [38]. Among other bioactive molecule-related genes found in *B. laterosporus* B.O.D. are basiliskamide A and B, polyketide compounds exhibiting antifungal activity against *Aspergillus fumigatus* and *Candida albicans* [39]. According to our results, this antimicrobial profile appears effective against phytopathogens, suggesting its potential application as a biocontrol agent in agricultural contexts. Similarly, several *B. laterosporus* isolates are strongly active against various *Fusarium* species [40,41].

The genome of *B. laterosporus* B.O.D. harbors a wide range of genes encoding putative toxins, enzymes, and virulence factors associated with entomopathogenic activity. Genes encoding homologous spore coat and canoe-shaped parasporal body (SC-CSPB) complex proteins were found in the genome, specifically spore surface protein A (CpbA) and exosporium protein C (ExsC). Both proteins were reported by Marche et al. [42] as virulence factors contributing to the insecticidal action of *B*. *laterosporus* strain UNISS 18 against the house fly. Multiple BLAST+ v2.17.0 (NCBI) analyses showed that strain B.O.D. also encodes several proteins sharing varying degrees of homology (85.10—100%) with other entomopathogenic *B. laterosporus* strains [17,43]. This set of proteins includes chitinase (ChiA), chitodextrinase (ChiD), collagenase-like protease (PrtC), thermophilic serine proteinase (Tsp), bacillolysin BL18 (Bl18), GlcNAc-binding protein A (Gbp), protective antigen domain protein (Pa1), and the insecticidal toxin Mpp75Ab1 [20]. Despite this similarity, the sequences of these homologous genes differed significantly, supporting variation in the insecticidal potential associated with different *B. laterosporus* strains.

Strain B.O.D. was found to be significantly active as an entomopathogen against the different insect species on which it was tested. While on one hand, this increases our knowledge of this strain, on the other, it is consistent with what is known in scientific literature on this bacterial species [2]. Accordingly, different strains are expected to exhibit varying degrees of toxicity against different targets. Hence, in our bioassays, strain B.O.D. generally showed activity comparable to strains NRS-661 and DSM25, while it was less active than strains UNISS18 and LMG 15441, which are known to be fully validated and characterized bioinsecticides [43,44,45]. Hence, bioassay results of *B. laterosporus* against Lepidoptera are inconsistent due to substantial variability among strains. Rivers et al. [46] reported that 28 *B. laterosporus* strains lacked pathogenic activity against the lepidopteran species *Ostrinia nubilalis* or *Manduca sexta*. In the present study, strain B.O.D. exhibited 53% and 75% mortality against *L. dispar* and *H. armigera*, respectively, at a concentration of 1 × 10^8^ spores/mL. On the other hand, *B. laterosporus* strain V12/001946, isolated from hybrid cabbage seed, exhibited a strong lethal effect against several Lepidopteran pests at a concentration of 10^10^ cells/mL [47]. Moreover, strain UNISS18 displayed a 100% mortality rate after 48 h when larvae of *Galleria melonella*, *L. dispar*, and *Malacosoma neustria* were injected with a suspension of exponential vegetative cells [48]. Initial evidence of the insecticidal properties of *B. laterosporus* came from studies on mosquitoes when Favret and Yousten [49] demonstrated the pathogenicity of 29 strains against larvae of *Culex quinquefasciatus*, *Aedes aegypti*, and the black fly *Simulium vittatum*. In the present study, strain B.O.D. exhibited approximately 20% lower toxicity than UNISS18 against various dipteran species. However, when comparing the different target species, the differences in mortality within the same strain (B.O.D. or UNISS18) were minimal, which supports the broad spectrum of activity known for this bacterium [2]. On the other hand, slight differences between target species might be expected in relation to specific factors such as insect physiology, gut environment, feeding behavior, and susceptibility to bacterial toxins.

It is noteworthy that the variation in bioinsecticidal potential of different *B. laterosporus* strains is consistent with the clustering patterns derived from genetic analyses, especially those based on comparisons of gene sequences involved in insecticidal activity. At the genetic level as well, strain B.O.D. shows greater similarity to strains NRS-661 and DSM25, which cluster closely together, whereas UNISS18 and LMG 15441 form a separate, closely related cluster that is more distant from B.O.D. Therefore, this comparative genetic analysis, integrated with the phenotypic data (bioinsecticidal potential), helps to characterize strain B.O.D. in terms of its versatility.

At present, there are no commercial products based on *B. laterosporus* being used as biopesticides. One aspect that makes it particularly interesting is its broad spectrum of activity, which represents a competitive advantage over other bacterial bioinsecticides that have specific modes of action against certain species but are ineffective against others [50]. Although the spectrum of activity is broad, its safety towards beneficial entomofauna and non-target organisms makes this bacterial species particularly promising [51]. Furthermore, the unique characteristics of the BOD strain, used as a probiotic in warm-blooded animals, constitute a further aspect of mitigating the risks normally associated with the use of plant protection products.

## 4. Conclusions

This study reports, for the first time, the genome of the probiotic *B. laterosporus* strain B.O.D., providing a comparative analysis and highlighting its insecticidal properties, which appear moderate relative to previously characterized entomopathogenic strains of the same species. Sequence alignment with other well-characterized *B. laterosporus* strains and analysis revealed significant differences, which support the corresponding differences in insecticidal activity observed when comparing strain B.O.D. with others against different lepidopteran and dipteran pest species. According to these findings, *B. laterosporus* strain B.O.D. appears to be remarkably versatile, underscoring its wide biotechnological potential. Among its potential applications, its antimicrobial activity against plant pathogens and its insecticidal properties make it suitable for managing a variety of targets.

## 5. Materials and Methods

### 5.1. Bacterial Strains and Culture Conditions

*Brevibacilus laterosporus* strain B.O.D. was isolated from the commercial product Flora-Balance^TM^ Powder (O’Donnell Formula Inc., San Marcos, CA, USA). Strain UNISS18 (=NCIMB41419) is maintained in the culture collection of the University of Sassari (Italy) [20]. Strains LMG 15441 (=ATCC 9141), NRS-661 (=ATCC 6456), and DSM25 (=ATCC 64) were originally provided by the Bacillus Genetic Stock Center (BGSC, Columbus, OH, USA).

Bacteria were routinely cultured in LB agar at 30 °C to maintain pure colonies. Liquid cultures were prepared in conical flasks containing LB broth at 30 °C while shaking at 180 rpm. To facilitate culture synchronization, an LB broth pre-culture at the exponential phase, started with heat-activated spores, was inoculated into CCY sporulation medium as described in Ruiu et al. [14]. Pure spore suspensions used in bioassays were harvested and washed by centrifugation before being serially diluted and quantified by counting the number of colony-forming units (CFU) on LB agar plates, according to Marche et al. [20]. Bacterial suspensions were adjusted with sterile water to achieve the concentration required for the bioassays.

### 5.2. Sequencing of B. laterosporus Strain B.O.D. Genome

DNA from *B. laterosporus* strain B.O.D. was extracted from a pure culture using the DNeasy Blood and Tissue Kits (Qiagen, Hilden, Germany) according to the manufacturer’s instructions. Genome analysis was performed using long-read sequencing data generated with Oxford Nanopore Technologies (Oxford, UK) (ONT) and carried out by Eurofins Genomics (Ebersberg, Germany) according to the following pipeline: Raw reads were quality-filtered using Filtlong v0.2.1 [52] to remove low-quality and very short reads, retaining only high-quality sequences for further analysis. Read quality metrics, including length distribution and quality score profiles, were assessed using NanoPlot v1.40.2 [53]. To optimize assembly performance and computational efficiency, reads were further downsampled using Filtlong v0.2.1. De novo genome assembly was conducted with Flye v2.9.3 [54], and the resulting assembly was polished with Medaka v1.8 [55] to improve base-level accuracy. Quality-filtered and downsampled reads were then aligned back to the polished assembly using Minimap2 v2.28 [56] to support downstream analyses. Variant calling was conducted using Clair3 v1.0.4 [57]. Detected variants, including SNPs and indels, were filtered and incorporated into the assembly using bcftools v1.10.2 [58], generating a high-confidence consensus sequence. Bakta v1.8.2 [59] was used for genome annotation so as to identify coding sequences, rRNAs, tRNAs, and other genomic features. Assembly quality was evaluated with QUAST v5.2.0 [60] to assess metrics such as N50, total genome length, and GC content, alongside CheckM2 v1.0.1 [61] to estimate genome completeness and contamination. Mash v2.3 [62] was used for comparative genomic analysis and taxonomic placement, calculating genomic distances against reference databases. Circular genome visualization was generated with CGView v2.0.3 [63], illustrating genomic features, GC skew, and coverage. Secondary metabolite biosynthesis gene clusters were predicted using the antiSMASH online platform version 8.0.2. [24].

### 5.3. Comparative Analysis

To perform genome-level comparative analyses, the genome sequences of nine *B. laterosporus* strains with documented insecticidal activity were retrieved from the NCBI database (Table A3). Average Nucleotide Identity (ANI) was calculated using FastANI (version 1.3) [64] on the European Galaxy server [65] with the B.O.D. genome as the reference. The distance matrix derived from ANI comparisons was used to construct a phylogenetic tree using the Neighbor-Joining method implemented in MEGA version 12.0.14 [27].

### 5.4. Multi-Gene Phylogeny

Gene prediction and annotation were performed with Bakta (Bakta v1.8.2 DB: v5.0—Light Proksee tool version 1.1.0) [60]. The genome was subsequently analyzed to identify genes associated with potential insecticidal activity based on available literature data. To assess the degree of homology, sequence alignments were conducted between selected B.O.D. genes related to insecticidal properties and those of all strains included in this study using the NCBI BLAST+ 2.17.0 suite [66]. A set of 13 genes (Table A2) was concatenated and used to construct a multi-gene phylogenetic tree using the Maximum Likelihood method and the Tamura–Nei model in MEGA version 12.0.14 [25,26] with 1000 bootstrap replicates.

### 5.5. Insect Bioassays

A group of experiments was conducted to assess the lethal effects of *B. laterosporus* strain B.O.D. on insects from different orders, including Lepidoptera and Diptera, in comparison with *B. laterosporus* strain UNISS18, well-known for its insecticidal potential.

Larvae of the gypsy moth *Lymantria dispar* L. (Lepidoptera: Erebidae) and of the cotton bollworm *Helicoverpa armigera* Hübner (Lepidoptera: Noctuidae) were field-collected and maintained in the laboratory on fresh cork oak and tomato leaves, respectively, until reaching the third instar. These larvae, being acclimatized to laboratory conditions, were then directly used in bioassays.

Adults of the house fly *Musca domestica* L. (Diptera: Muscidae) and of the green bottle fly *Lucilia caesar* (Diptera: Calliphoridae) were provided by the entomology laboratory of the Department of Agricultural Sciences of the University of Sassari (Italy). Eggs of the Asian tiger mosquito *Aedes albopictus* Skuse (Diptera: Culicidae) and of the common house mosquito *Culex pipiens* L. (Diptera: Culicidae) were provided by the Zooprophylactic Institute of Sardinia (Italy). All Diptera specimens were derived from colonies established in the laboratory several years earlier.

Bioassays were conducted under controlled conditions in a bioassay room at 25 °C with a 14:10 h (light–dark) photoperiod.

In the case of *L. dispar* and *H. armigera*, groups of third instar larvae (*n* = 10) were kept inside sterile Petri dishes and provided ad libitum with fresh leaves of *Quercus suber* L. or tomato, respectively, previously treated by spraying with bacterial spore suspension at a concentration of 10^8^ spores/mL or with water only (control), according to Ruiu et al. [48]. Insects were then inspected daily, and mortality was recorded. The experimental design involved four replicates and was repeated twice. For *M. domestica* and *L. caesar*, newly emerged fly adults (*n* = 10) were instead reared inside transparent boxes (10 × 10 × 10 cm) where liquid food was provided by two 50 μL capillary tubes refreshed daily (10 μL/fly/day). This liquid consisted of a 30% sucrose solution containing 10^8^ bacterial spores/mL (treated) or plain juice (control). Cages were inspected daily for 72 h to record insect mortality.

Mosquito bioassays were conducted with third instar larvae that were maintained in groups of 10 inside plastic cups containing 50 mL sterile water with 10^6^ spores/mL (treated) or left untreated (control). Cups were maintained inside an incubator at 25 °C for daily inspections, and mortality was assessed after 48 h. The experimental design involved four replicates and was repeated three times.

Additional experiments were conducted with *M. domestica* adults according to the previously described protocol to compare the lethal effects determined by different *B. laterosporus* strains (B.O.D., UNISS18, LMG15441, NRS-661, and DSM25) when exposed to a standard concentration of spores (10^8^/mL).

In another experiment, following the same methodology, house fly adults were instead exposed to a higher (10^9^ spores/mL) and a lower (10^7^ spores/mL) concentration of *B. laterosporus* strain B.O.D. spores, and mortality was assessed daily in comparison with a control.

Another experiment was conducted with *A. albopictus* following the previously described protocol to determine the median lethal concentration (LC_50_) of *B. laterosporus* strain B.O.D. spores. For this purpose, third instar larvae were exposed to the following range of concentrations: 1.0 × 10^6^, 0.5 × 10^6^, 0.25 × 10^6^, 0.1 × 10^6^, 0.5 × 10^5^, 0.25 × 10^5^, and 0.1 × 10^5^ spores/mL.

All the concentrations selected for testing were based on previous knowledge of the activity of other *B. laterosporus* strains against the various targets, which has enabled comparisons to be made with the B.O.D. strain under investigation in this study [2].

### 5.6. Antifungal Bioassays

A set of bioassays was conducted to evaluate the antimicrobial properties of *B. laterosporus* strain B.O.D. in comparison with strain UNISS18 against phytopathogenic fungi, including *Fusarium graminearum*, *F. culmorum*, and *F. verticillioides*, obtained from the Laboratory of Plant Pathology (University of Sassari, Italy). The fungal pathogens were preliminarily cultured on 90 mm diameter potato dextrose agar (PDA; VWR International, Radnor, PA, USA) at 25 °C for 7 days. Bacteria were streaked onto the center of new PDA plates and incubated at 28 °C for 48 h. Two agar plugs from 7-day-old fungal cultures were positioned perpendicular to the bacterial streak, 1.5 cm from the plate edge. Co-cultures were incubated in a growth chamber at 28 °C for 7 days. Fungal colonies were photographed, and images were taken on day 5 and analyzed using ImageJ v1.54 (NIH, Bethesda, MD, USA) to calculate colony diameter (mm) and area [67].

### 5.7. Statistical Analysis

R software, version 4.4.3, was used for statistical analysis [68]. One-way analysis of variance (ANOVA) (factor: treatment) followed by a Tukey post hoc test [69] was used to analyze insect mortality data. The assumption of homogeneity of variance across groups was verified by Levene’s test. Insect survival over time was analyzed using a linear mixed-effects model (LMM) to account for repeated measures. The model was fitted using restricted maximum likelihood (REML), and estimated marginal means (EMMs) with Tukey-adjusted pairwise comparisons were computed to assess group differences. The relationship between mosquito larval mortality and bacterial concentration was assessed using linear regression, while probit regression with a binomial error distribution and log-transformed concentration as a predictor was used to calculate the median lethal concentration (LC_50_). The effects of bacterial strains and fungal species on the area of mycelial growth on plates were assessed by two-way ANOVA followed by Tukey’s post hoc test for pairwise comparisons.

## Figures and Tables

**Table 1:** Comparative efficacy (mortality ± SE) of *Brevibacillus laterosporus* strains B.O.D. and UNISS18 against insect species in two orders.

Insect Species	Mortality ^1^ (±SE)	
	Strain B.O.D. ^2^	Strain UNISS18 ^2^
Lepidoptera		
*Lymantria dispar*	53.2 ± 2.7 a ^3^	73.4 ± 4.3 b
*Helicoverpa armigera*	74.7 ± 1.9 a	92.4 ± 2.5 b
Diptera		
*Musca domestica*	63.3 ± 2.7 a	88.6 ± 2.3 b
*Lucilia caesar*	58.2 ± 3.0 a	72.2 ± 2.5 b
*Aedes albopictus*	77.2 ± 2.5 a	91.1 ± 2.3 b
*Culex pipiens*	89.9 ± 2.7 a	98.7 ± 1.2 b

^1^ Mortality was calculated at 72 h for *L. dispar*, *H. armigera*, *M. domestica*, and *L. caesar*, and at 48 h for *A. albopictus* and *C. pipiens*. ^2^ Bacteria were administered at a concentration of 1 × 10^8^ spores/mL to *L. dispar*, *H. armigera*, *M. domestica*, and *L. caesar* and of 1 × 10^6^ spores/mL to *A. albopictus* and *C. pipiens*. ^3^ Different letters in a row indicate significantly different means (1-way ANOVA, followed by Tukey test, *p* < 0.05). Mortality in the control was always below 5%.

## Data Availability

Bacterial genomes and annotated genes are deposited in GenBank, and accession numbers are provided in the text.

## Abbreviations

The following abbreviations are used in this manuscript:

PDA	Potato Dextrose Agar
LB	Luria Bertani
ANIE	Average Nucleotide Identity
ONT	Oxford Nanopore Technologies
LC	Lethal Concentrations
ANOVA	Analysis of Variance
EMMs	Estimated Marginal Means
LMM	Linear Mixed-Effects Model
REML	Restricted Maximum Likelihood

## Appendix A

**Table A1:** Percentage of Average Nucleotide Identity (ANI) in *Brevibacillus laterosporus* strains, calculated using FastANI.

	B.O.D.	DSM_25	SN19_1	SAM19	Wq_1	BON707	LMG_ 15441	UNISS18	GI-9	NRS-661
**B.O.D.**		98.9401	99.9925	99.1211	99.9948	89.3488	89.4418	89.2824	89.2949	99.0022
**DSM_25**			98.8955	98.8113	98.9024	89.3059	89.3849	89.3792	89.2409	98.9142
**SN19_1**				99.1394	99.9921	89.3089	89.4074	89.2604	89.2471	99.0001
**SAM19**					99.1613	89.3299	89.302	89.2626	89.3261	98.9106
**Wq_1**						89.3178	89.4375	89.2593	89.2961	99.0032
**BON707**							99.9695	98.6637	98.4252	89.2813
**LMG_15441**								98.6318	98.398	89.2754
**UNISS18**									98.3418	89.4306
**GI-9**										89.2995
**NRS-661**										

**Table A2:** Selection of insecticidal genes in *Brevibacillus laterosporus* strain B.O.D. and their sequence homology across related strains. The first column lists genes associated with insecticidal activity, followed by their accession numbers in strain B.O.D. Subsequent columns report the percentage of sequence homology of each gene in the corresponding strains relative to strain B.O.D.

Gene	Accession No (Strain B.O.D.)	Other Strains (% Identity)								
		Wq-1	SN19-1	SAM19	NRS-661	DSM 25	GI-9	UNISS18	BON707	LMG 15441
Chitinase A (*chiA*)	PZ232209	100.00	100.00	100.00	99.74	99.84	90.14	89.62	89.83	89.83
Chitodextrinase (*chiD*)	PZ232210	100.00	100.00	99.61	99.92	99.53	88.50	88.50	88.43	88.43
Collagenase-like protease (*PrtC*)	PZ232211	100.00	100.00	99.84	99.92	99.92	94.61	94.30	94.30	94.30
Thermophilic serine proteinase (*tsp*)	PZ232212	100.00	100.00	100.00	99.73	99.79	87.73	87.66	87.86	87.86
Spore surface protein A (*cpbA*)	PZ232213	100.00	100.00	99.74	99.74	99.74	90.89	90.89	90.89	90.89
Exosporium protein C (*exsC*)	PZ232214	100.00	100.00	100.00	99.07	98.61	87.96	88.19	88.19	88.19
Gramicidin S synthase 1 (*grsA*)	PZ232215	100.00	100.00	99.81	99.13	98.94	85.95	85.49	85.77	85.82
Bacillolysin BL18 (*bl18*)	PZ232216	100.00	100.00	99.88	99.88	99.88	91.44	91.38	91.20	91.20
Surfactin synthase subunit 1 (*srfAA*)	PZ232217	100.00	100.00	99.71	99.68	99.68	85.10	85.42	85.29	85.29
GlcNAc-binding protein A (*gbp*)	PZ232218	100.00	100.00	100.00	99.52	99.66	89.80	89.73	89.46	89.46
Hexagonal wall protein (*hwp*)	PZ232219	100.00	100.00	99.87	98.69	99.53	93.59	94.25	94.09	94.06
Protective antigen domain protein (*pa1*)	PZ232220	98.78	98.62	94.48	96.55	97.30	86.86	87.03	87.19	87.19
*Mpp75Ab1*	ON007247.1	99.00	99.00	98.00	98.32	98.54	98.42	98.32	98.21	98.21

**Table A3:** *Brevibacillus laterosporus* strains used in this study for comparative genomic analysis. Genome sequences were retrieved from the NCBI database and used as references for comparison with strain B.O.D. investigated in this study.

*Brevibacillus laterosporus* Strain	Accession No.
DSM 25 (=ATCC64)	ASM270679v1
SN19-1	ASM4152996v1
SAM19	ASM1690490v1
Wq-1	ASM3319267v1
BON707	ASM359476v1
LMG 15441 (=ATCC9141)	ASM21953v3
UNISS18	ASM169670v1
NRS-661 (=ATCC6456)	ASM5047598v1
GI-9	ASM23700v2
